# Transcriptomic and Metabolomic Response to High Light in the Charophyte Alga *Klebsormidium nitens*

**DOI:** 10.3389/fpls.2022.855243

**Published:** 2022-05-06

**Authors:** Emma Serrano-Pérez, Ana B. Romero-Losada, María Morales-Pineda, M. Elena García-Gómez, Inmaculada Couso, Mercedes García-González, Francisco J. Romero-Campero

**Affiliations:** ^1^Microalgae Systems Biology and Biotechnology Research Group, Institute for Plant Biochemistry and Photosynthesis, Universidad de Sevilla – Consejo Superior de Investigaciones Científicas, Seville, Spain; ^2^Department of Computer Science and Artificial Intelligence, Universidad de Sevilla, Seville, Spain

**Keywords:** light stress, Charophyta, omics integration, plant evolution, carotenoids, chloroplast retrograde signaling, linear/cyclic electron flow, PsbS/LHCSR NPQ systems

## Abstract

The characterization of the molecular mechanisms, such as high light irradiance resistance, that allowed plant terrestralization is a cornerstone in evolutionary studies since the conquest of land by plants played a pivotal role in life evolution on Earth. Viridiplantae or the green lineage is divided into two clades, Chlorophyta and Streptophyta, that in turn splits into Embryophyta or land plants and Charophyta. Charophyta are used in evolutionary studies on plant terrestralization since they are generally accepted as the extant algal species most closely related to current land plants. In this study, we have chosen the facultative terrestrial early charophyte alga *Klebsormidium nitens* to perform an integrative transcriptomic and metabolomic analysis under high light in order to unveil key mechanisms involved in the early steps of plants terrestralization. We found a fast chloroplast retrograde signaling possibly mediated by reactive oxygen species and the inositol polyphosphate 1-phosphatase (SAL1) and 3′-phosphoadenosine-5′-phosphate (PAP) pathways inducing gene expression and accumulation of specific metabolites. Systems used by both Chlorophyta and Embryophyta were activated such as the xanthophyll cycle with an accumulation of zeaxanthin and protein folding and repair mechanisms constituted by NADPH-dependent thioredoxin reductases, thioredoxin-disulfide reductases, and peroxiredoxins. Similarly, cyclic electron flow, specifically the pathway dependent on proton gradient regulation 5, was strongly activated under high light. We detected a simultaneous co-activation of the non-photochemical quenching mechanisms based on LHC-like stress related (LHCSR) protein and the photosystem II subunit S that are specific to Chlorophyta and Embryophyta, respectively. Exclusive Embryophyta systems for the synthesis, sensing, and response to the phytohormone auxin were also activated under high light in *K. nitens* leading to an increase in auxin content with the concomitant accumulation of amino acids such as tryptophan, histidine, and phenylalanine.

## Introduction

The evolutionary history of the green plants kingdom or Viridiplantae splits into two different lineages Chlorophyta and Streptophyta. Chlorophyta are primarily constituted by marine and freshwater green microalgae. In turn, Streptophyta are divided into two different clades Charophyta and Embryophyta. Whereas Embryophyta comprises mainly land plants, Charophyta are still considered algae with a preference for freshwater and with some facultative terrestrial species (Becker and Marin, 2009). It is widely accepted that the primary adaptation of Charophyta to freshwater played a key role facilitating their transition to dry land in contrast to marine Chlorophyta. Plant terrestralization constitutes a key milestone in life evolution on Earth since it led to a massive increase in land biomass resulting in a substantial atmospheric CO_2_ drop, oxygen increase and terrestrial habitat stabilization promoting land colonization by animals and fungi (Lenton et al., 2016; Morris et al., 2018). Present-day Charophyta are generally accepted as the extant algal species most closely related to the aquatic ancestors of land plants or Embryophyta. Accordingly, the molecular systems that potentially allowed this group of photosynthetic organisms to evolve toward terrestrial land plants are under intense analysis (Nishiyama et al., 2018). These studies focus mainly on genomic data. The lack of multi-omic data such as transcriptomic and metabolomic data for Charophyta under specific conditions relevant to the terrestralization process is preventing the full characterization of the molecular systems that promoted the transition to the first land plants (Hori et al., 2014). During this transition, the evolution of response molecular systems to terrestrial environmental stresses was critical. Some terrestrial physiological adaptations, such as desiccation resistance and tolerance to UV radiation are present in Charophyta from which current land plant mechanisms supposedly evolved (Becker and Marin, 2009). Multiple cellular features such as phragmoplast, plasmodesmata, hexameric cellulose synthase, and oogamous sexual reproduction with zygote retention first evolved in Streptophytic algae or Charophyta leading to multicellularity (Umen, 2014). A tight retrograde signaling communicating the chloroplast state to the nucleus making plastids more dependent on the nucleus has been reported in Charophyta under cold and high light stresses (De Vries et al., 2018). Other systems found in Embryophyta such as abscisic acid (ABA) and auxin biosynthesis and transport, photoprotective capacity, and adaptation to transient light changes have been identified in Charophyta as *Zygnema circumcarinatum* (Ohtaka et al., 2017; Pierangelini et al., 2017). Biosynthetic pathways sources of metabolites relevant to abiotic stresses typical of terrestrial environments such as the phenylpropanoid pathway has been described to first emerged in Charophyta (de Vries et al., 2021). In this study, we have chosen the freshwater facultative terrestrial Charophyte alga *Klebsormidium nitens* (*K. nitens*) as model organism to study the transcriptomic and metabolomic response to high light intensity recreating at least one of the most critical environmental changes faced by plants during terrestralization. *Klebsormidium* cultures consist of multicellular and non-branching filaments without specialized cells with a single chloroplast. Many *Klebsormidium* species are cosmopolitan distributed in terrestrial environments as soil crusts and rocks as well as freshwater habitats like streams and rivers where they contribute to important ecological roles as primary producers and soil stabilizers (Karsten et al., 2016). Their presence in these environments expose cells to extreme conditions including high light irradiance (Holzinger and Pichrtová, 2016). Physiological studies under such conditions have been carried out reporting photosynthetic resistance against intense light meditated by the presence of photoprotective mechanisms dissipating energy as heat (non-photochemical quenching, NPQ) (Gerotto and Morosinotto, 2013) and/or by the activation of alternative electron routes to reduce reactive oxygen species (ROS) production (Alboresi et al., 2019). Several comparative genomic analyses have been carried out providing evidence about *K. nitens* possessing fundamental molecular mechanisms required for the adaptation and survival in terrestrial environments including wax-related genes (Kondo et al., 2016), phytohormone signaling (Holzinger and Becker, 2015), and transcription factors involved in resistance to high light and UV radiation (Kitzing and Karsten, 2015; Domozych et al., 2016). Nonetheless, there are very few transcriptomic studies integrating gene expression with physiological data aiming at the characterization of *K. nitens* responses to abiotic stresses such as desiccation, cold, and heat (Holzinger et al., 2014; Rippin et al., 2019a; de Vries et al., 2020; Monte et al., 2020). Furthermore, *K. nitens* is also of interest for its biotechnological applications in the removal of nutrients from horticultural wastewater (Liu et al., 2016a) and in the production of polyunsaturated fatty acids and lipids (Liu et al., 2016b; Xu et al., 2021).

The goal of the current study consists in identifying the molecular mechanisms underlying the response to high light intensity in the Charophyte alga *K. nitens*. The similarity of these systems with those used by Embryophyta and Chlorophyta is discussed in order to elucidate the key mechanisms that allowed the transition from aquatic environments to dry land during plant evolution. Our results were obtained from an integrative analysis combining gene expression and metabolite profiles. Further validation of our results were carried out using pulse-amplitude-modulation fluorometry (PAM), Western blotting, and confocal microscopy.

## Materials and Methods

### Algal Material, Growth Conditions, and Sample Collection

*Klebsormidium nitens* (strain NIES-2285) was obtained from the National Institute for Environmental Studies (Japan). Cells were grown photoautotrophically in Bold’s Basal Medium using photobioreactors containing 0.8 L of cell suspension and bubbled with air supplemented with 1% (v/v) CO_2_ as carbon source. Photobioreactors were continuously illuminated with white light lamps at 50 μE m^–2^ s^–1^ and maintained at 20°C. Defoamer (Antifoam 204) was added to avoid the contamination of the aeration systems. Cultures at exponential phase with 45 μg/mL chlorophyll content were used in our experiments. Control cultures were kept under a light irradiance of 50 μE m^–2^ s^–1^ whereas high light cultures were illuminated for 3 h with an irradiance of 1500 μE m^–2^ s^–1^. Cells were collected by centrifugation at 3500 × *g* for 5 min at 4°C. Cell pellets were washed with PBS, flash frozen with liquid Nitrogen and stored at −80°C.

### RNA-Seq Data Generation and Processing

Two independent biological replicates were considered for both low and high light irradiance conditions. RNA extraction was performed using mechanical disruption of the frozen cell pellets in a Mini Bead Beater (Biospe Products) mixed with 2.7 mm glass beads for filament fragmentation and 0.5 mm glass beads for individual cell lysis (ratio 1/3) in the presence of an extraction buffer consisting of phenol:chloroform (1:1, v/v). Subsequently, RNA was purified using ISOLATE II RNA Plant Kit (Bioline) following manufacturer’s instructions. RNA integrity number (RIN) was computed using an Agilent 2100 Bioanalyzer producing values greater than 8 per sample. Sequencing libraries were generated according to Illumina TruSeq Stranded mRNA protocol and sequenced on an Illumina NextSeq 500 sequencer producing approximately 17 million 50 nt long reads per sample. The computational pipeline MARACAS (Romero-Losada et al., 2022) was used to determine differentially expressed genes according to a log2FC of ±1 and a *q*-value or false discovery rate (FDR) threshold of 0.05. MARACAS uses the *K. nitens* genome sequence assembly and annotation v1.0 (accession number DF236950) as reference genome (Hori et al., 2014). The software tool AlgaeFUN^1^ was used to perform functional enrichment analysis based on Gene Ontology (GO) terms and Kyoto Encyclopedia of Genes and Genomes (KEGG) pathways over the sets of differentially expressed genes.

Specifically, in our study, MARACAS was set to run using fastqc, HISAT2 and Stringtie for quality control, read mapping, transcript assembly, and gene expression quantification, respectively (Pertea et al., 2016). Normalization is carried out in MARACAS based on the Bioconductor R package NormalyzerDE (Willforss et al., 2019). In our study, gene expression was normalized using quantile normalization. Differentially expressed genes were determined in MARACAS using the bioconductor R package limma based on linear models with a moderated t-student (Ritchie et al., 2015). This method estimates individual gene expression variance using information from all genes and can be applied in analysis with two replicates in contrast to R packages such as DESeq2 based on binomial negative distribution that require at least three replicates (Love et al., 2014). Several studies comparing the performance of limma (linear models with a moderated *t*-student) and DEseq2 (negative binomial distribution) conclude that, although they are mostly equivalent, limma outperforms DESeq2 at reducing batch effects and false positives (Seyednasrollah et al., 2013; Stupnikov et al., 2021). The presence of low levels of noise in our two replicates was confirmed in MARACAS using scatterplots and principal components analysis (PCA), Supplementary Figure 1. We also checked that our sequencing coverage (or depth) with more than 17 million reads per sample was enough to detect gene expression and determine differentially expressed genes using a saturation analysis that identified around 12 million read as the saturation point for RNA-seq data in *K. nitens* (Supplementary Figure 1). In conclusion, the read coverage, level of noise in our data and use of linear models with a moderated *t*-student would guarantee the reliability of our analysis based on two replicates.

### Metabolomic Data Generation and Processing

Six independent biological replicates were considered for low and high light irradiance metabolomic data generation. Significant differences were determined using the non-parametric Wilcoxon signed-rank test implemented in the wilcox.test function from the stats R package.

For metabolite content determination, cell pellets were lyophilized (Skadi-Europe TFD 8503), flushed with a nitrogen stream to prevent oxidation and stored at −20°C. Primary metabolites were determined from 20 mg of lyophilized biomass subjected to mechanical disruption in a Mini Bead Beater (Biospe Products) with a mixture of 2.7 and 0.5 mm glass beads (ratio 1/3) in the presence of 1 mL extraction buffer consisting of chloroform:methanol (3:7, v/v). As internal standard, 5 μL of ribitol 4 mM were added. Following centrifugation at 5000 × *g* for 5 min at RT (room temperature) the supernatant was collected. This process was repeated adding 1 mL of extraction buffer until the supernatant was colorless. The combined supernatants were dried under nitrogen stream, resuspended in Milli-Q water and submitted for analysis. Primary metabolite determination was carried out by ultra high performance liquid chromatography system coupled with mass spectrometry (UPLC/MS) as described in Mccloskey and Ubhi (2015).

Phytohormone content was determined from 50 mg of lyophilized biomass following the protocol presented in Salem et al. (2020). Cellular lysis and sample homogenization was performed as described above for RNA and primary metabolite extraction using, in this case, 1 mL of an extraction buffer consisting of methyl tert-butyl ether (MTBE):methanol (3:1, v/v). Samples were incubated for 30 min at 4°C in a rotating mixer, followed by sonication for 15 min at 4°C and centrifugation at 10,000 × *g* for 10 min. The supernatant was mixed with 0.1% HCl (1:1, v/v) and 20 μL of paracetamol added as internal standard at 4°C. Subsequently, samples were first vigorously vortexed for 1 min and then gently shaken in a rotating mixer for 30 min at 4°C and centrifuged again. The supernatant was dried overnight in spin vacuum and finally resuspended in water:methanol (1:1, v/v) and filtered for determination using UPLC/MS.

Carotenoid content was determined by high-performance liquid chromatography (HPLC) coupled to an UV-visible scanning spectrophotometer from 5 mg of lyophilized biomass using acetone extracts subjected to mechanical disruption for cell lysis and sample homogenization as described in Del Campo et al. (2004).

### Pulse-Amplitude-Modulation Fluorometry

Photosynthetic parameters were determined by pulse-amplitude-modulation fluorometry (PAM) with a DUAL-PAM-100 (Walz). Samples were dark adapted for 10 min before fluorescence was measured. Values for basal fluorescence level, F_0_, were determined after 5 min in the presence of non-actinic light 450 nm. Values for maximal fluorescence (F_m_) were determined by applying a pulse of saturating red light 655 nm, 2.4 μE for 40 s. Values for photosystem II (PSII) maximal efficiency (F_v_/F_m_) were calculated as (F_m_–F_0_)/F_m_. Cyclic electron flow (CEF) was detected by subjecting dark adapted samples to constant actinic light (500 μE) for 5 min and subsequently turning off light and measuring fluorescence.

### Total Protein Extraction, Sodium Dodecyl Sulfate-Polyacrylamide Gel Electrophoresis, and Western Blotting Analysis

Cells were pelleted by centrifugation at 3500 × *g* for 5 min at 4°C, washed with PBS and resuspended in 50 mM Tris pH 8, SDS 9%, PID, PMSF 0.1 M and NaCl 150 mM. Total protein extracts were obtained by freeze/thaw cycles in liquid nitrogen followed by another mechanical disruption procedure as described above. Protein extracts were separated by sodium dodecyl sulfate-polyacrylamide gel electrophoresis (SDS-PAGE), stained with Comassie blue, and transferred onto a PVDF membrane (Immobilon ^®^-P, pore size 0.45 μm) using a power blotting station (Invitrogen™, Thermo Fischer). Immunoblotting analysis was performed with an antibody against photosystem II subunit S (PsbS) at 1:1000 (Agrisera). Anti-rabbit secondary antibody was used at 1:10,000 (Invitrogen). Immunoblots were visualized using IQ800 Control software (ImageQuant 800, Amersham).

## Results and Discussion

### Transcriptomic and Metabolomic Analysis Unveil a Response to High Light Intensity

**FIGURE 1:**
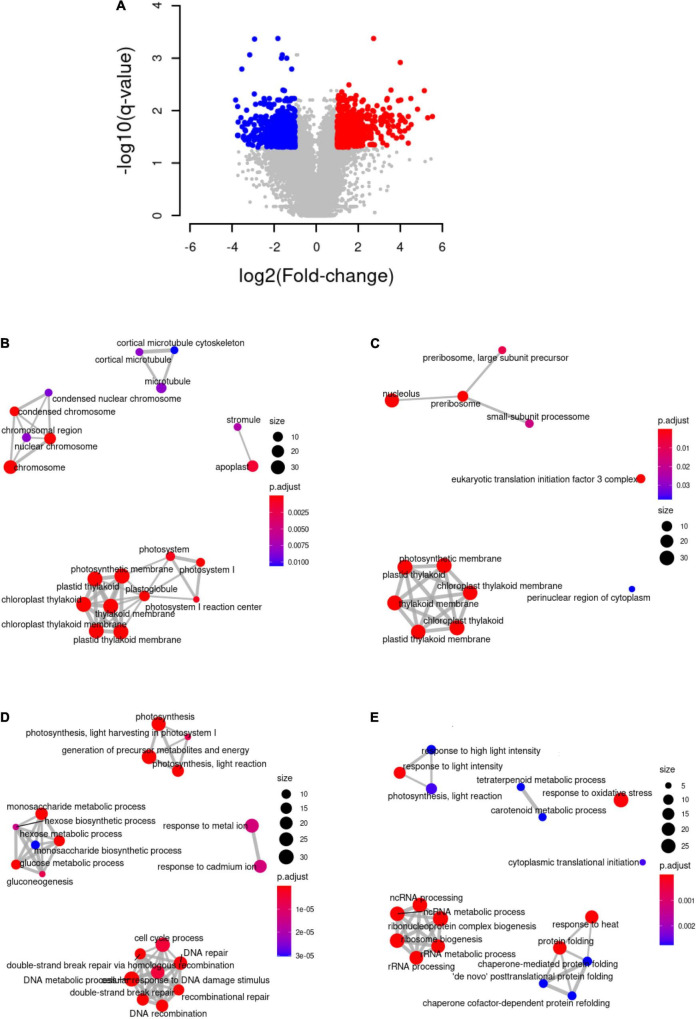
Differential gene expression analysis under high light intensity and functional enrichment analysis. **(A)** Volcano plot representing differentially activated (red), repressed (blue), and unaltered (gray) genes under high light when compared to low light. **(B)** Cellular components to which the proteins encoded by repressed genes are significantly associated. **(C)** Cellular components to which the proteins encoded by the activated genes are significantly associated. **(D)** Biological processes in which the proteins encoded by the repressed genes are significantly involved. **(E)** Biological processes in which the proteins encoded by the activated genes are significantly involved.

Nuclear gene expression responses to high light in *K. nitens* were studied using RNA-seq data. We detected expression in 68.4% of the 17,290 genes in the current *K. nitens* genome annotation (Hori et al., 2014). We found that after 3 h of high light treatment 7.84% of the entire *K. nitens* genome was differentially expressed with respect to low light conditions. Specifically, we identified 677 activated and 678 repressed genes (Figure 1 and Supplementary Table 1). Using AlgaeFUN (microALGAE FUNctional enrichment tool), we performed functional enrichment analysis based on GO terms to identify the cellular components and biological processes significantly affected by high light (Figure 1). The proteins encoded by differentially expressed genes, both activated and repressed genes, were significantly localized in the chloroplast thylakoid membranes indicating the initiation of a major chloroplast reprogramming. Specifically, proteins encoded by repressed genes were significantly associated with photosystems and cellular structures present during cell division such as condensed nuclear chromosomes and microtubules. Accordingly, photosynthesis, hexose biosynthesis, cell cycle, and DNA metabolism were significantly enriched processes in the repressed genes. This points to an arrest in the photosynthetic machinery and cell cycle progression as response to high light. Proteins encoded by activated genes are, in turn, significantly localized in cellular structures involved in *de novo* protein biosynthesis such as preribosomes and translation initiation factor 3′ complex. In particular, categories encompassing ribosome biogenesis, cytoplasmic translation initiation, and protein folding were significantly enriched in the activated genes. Moreover, response to oxidative stress, response to high light intensity, tetraterpenoid and carotenoid metabolism were identified as significantly activated processes. This suggests an activation of repair and protective mechanisms to damages caused by high light.

**FIGURE 2:**
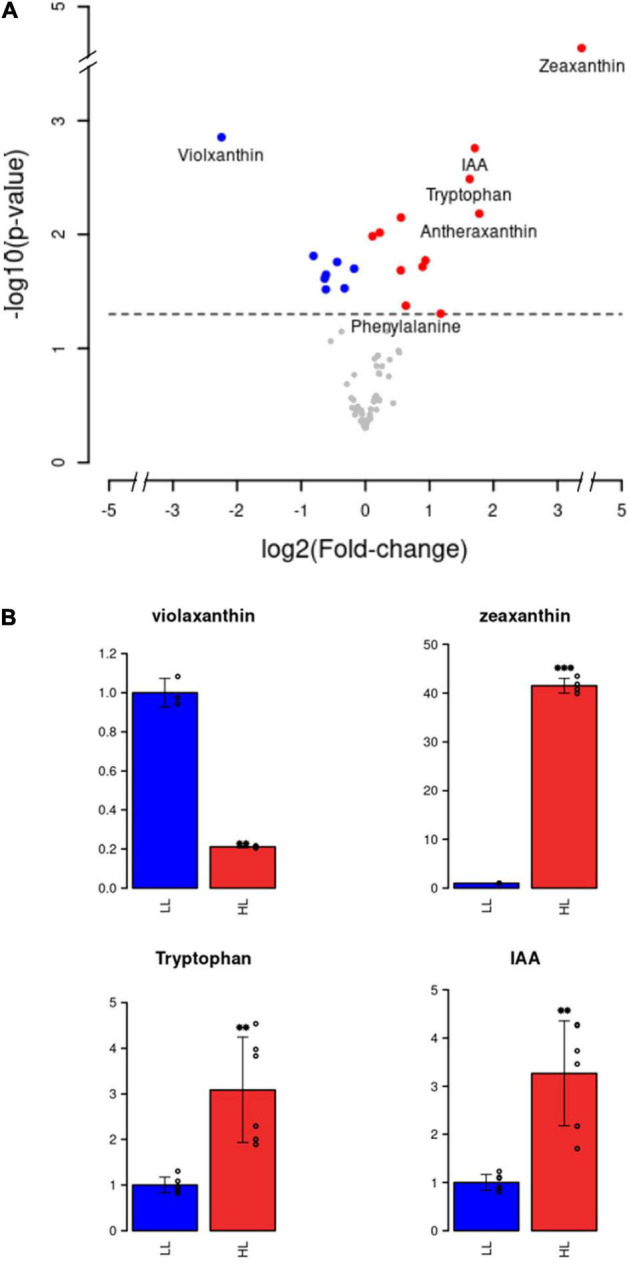
Differential metabolite abundance analysis under high light intensity. **(A)** Volcano plot representing more abundant (red), less abundant (blue), and equally abundant (gray) metabolites under high light when compared to low light. **(B)** Barplots representing the relative metabolite abundance under high light in red (HL) with respect to low light in blue (LL) for violaxanthin, zeaxanthin, tryptophan, and indole-3-acetic acid (IAA).

Metabolomic responses to 3 h of high light treatment in *K. nitens* were also analyzed. Six independent biological replicates were considered for both, high and low light conditions. We detected 69 different primary and secondary metabolites including most amino acids and some phytohormones, Supplementary Table 2. Significant differentially abundant metabolites were identified by performing the non-parametric Wilcoxon test using a *p*-value threshold of 0.05. We found 12 significantly more abundant and 8 less abundant metabolites under high light when compared to low light (Figure 2). For instance, under high light, we detected significant changes in specific carotenoids, accumulation of the amino acid tryptophan and the phytohormone indole-3-acetic acid (IAA).

### An Activation of the Carotenoid Biosynthesis β-Branch and Xanthophyll Cycle Is Observed

**FIGURE 3:**
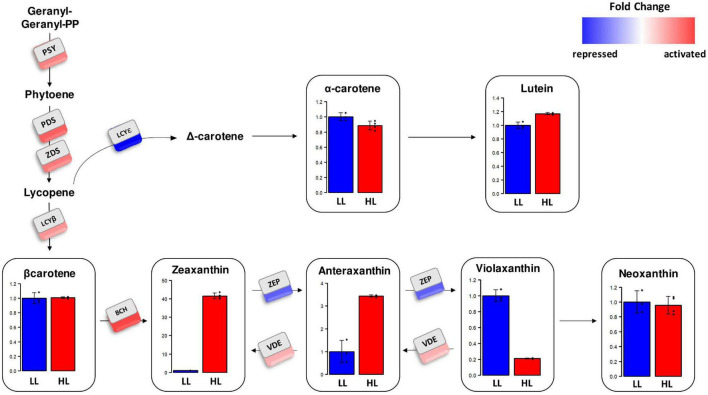
Gene expression level and relative carotenoid content in the carotenoid biosynthesis pathway in *Klebsormidium nitens* under high light (HL) and low light (LL). Expression fold change under high light compared to low light is represented for genes corresponding to the different enzymes involved in carotenogenesis in *Klebsormidium nitens*: phytoene synthase (PSY), phytoene desaturase (PDS), ζ-carotene desaturase (ZDS), lycopene epsilon cyclase (LCYε), lycopene beta cyclase (LCYβ), β-carotene hydroxylase (BCH), violaxanthin de-epoxidase (VDE), and zeaxanthin epoxidase (ZEP). Red squares represent strong activation whereas blue squares stand for strong repression. Barplots show relative carotenoid content under high light in red (HL) compared to low light in blue (LL).

Here, we present an integrated transcriptomic and metabolomic analysis of this specific photoprotective response to high light in *K. nitens* (Figure 3). The gene encoding the first enzyme in the carotenoid pathway and the main rate-limiting step, phytoene synthase (PSY, *kfl00019_0320*) was 1.53-fold activated after 3 h of high light treatment. Similarly, the genes encoding the next enzymes in the pathway producing lycopene, phytoene desaturase (PDS, *kfl00103_0130*), and ζ-carotene desaturase (ZDS, *kfl00496_0070*), were 1.88- and 1.64-fold activated, respectively. At this point carotenoid biosynthesis bifurcates into the ε-branch leading to lutein and the β-branch proceeding to β-carotene and the xanthophyll cycle. These two branches showed antagonist regulation in the response to high light in *K. nitens*. On the one hand, a strong gene repression of 7.49-fold was found for the enzyme funneling lycopene into the ε-branch, lycopene epsilon cyclase (LCYε, *kfl00536_0070*). Nonetheless, no significant change was observed in the carotenoids produced in this branch, α-carotene and lutein in contrast to the massive increase in this latest carotenoid observed in Chlorophyta as Chlamydomonas under high light (Ma et al., 2019). On the other hand, simultaneously, a strong gene activation of 2.52-fold was detected for the enzyme channeling lycopene into the β-branch, lycopene beta cyclase (LCYβ, *kfl00003_0600*) and of 2.24-fold for the enzyme β-carotene hydroxylase (BCH, *kfl00515_0050*) that converts β-carotene into zeaxanthin. This response has been also observed in Chlorophyta (Couso et al., 2012). Although, β-carotene content was similar under low and high light conditions, significant changes were found in the carotenoids constituting the xanthophyll cycle. Violaxanthin content decreased 4.73-fold whereas antheraxanthin and zeaxanthin contents were increased 3.44- and 41.5-fold, respectively, under high light when compared to low light. Accordingly, the gene encoding the enzyme involved in the xanthophyll cycle, violaxanthin de-epoxidase (VDE, *kfl00604_0070*) converting violaxanthin into antheraxanthin and zeaxanthin was activated 1.86-fold. Furthermore, the gene encoding zeaxanthin epoxidase (ZEP, *kfl00092_0060*) that catalyzes the synthesis of violaxanthin from zeaxanthin and antheraxanthin was 3.84-fold repressed under high light. In the xanthophyll cycle, the interconversion of violaxanthin into antheraxanthin and zeaxanthin, constitutes one of the major photoprotective mechanism in Embryophyta (Latowski et al., 2011) and Chlorophyta (Goss and Jakob, 2010). High light induces the mobilization of violaxanthin to zeaxanthin whereas low light or darkness produce the reverse reaction. De-epoxidation of violaxanthin to zeaxanthin enhances dissipation of excess excitation energy (NPQ) in the PSII antenna, thereby preventing inactivation and damage to the photosynthetic apparatus. NPQ is considered a fundamental mechanism for Streptophyta adaptation to terrestrial habitats (Pierangelini et al., 2017). Here, we specifically show that the xanthophyll cycle is part of the early transcriptomic and metabolomic response to high light intensity in the Charophyta *K. nitens*.

### Chloroplast Retrograde Signaling Triggered by Oxidative Stress and Protein Misfolding Is Identified as a Response to High Light

**FIGURE 4:**
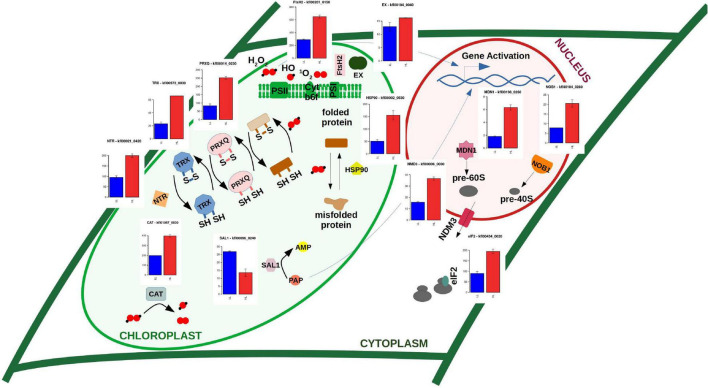
Gene expression level for enzymes involved in retrograde signaling triggered by high light oxidative stress inducing gene activation of protein repair mechanisms and *de novo* protein synthesis. Barplots represent gene expression measured in FPKM (Fragments per Kilobase of exon and million of Mapped reads) under high light in red (HL) and low light in blue (LL) for enzymes EX (executer), protease FtsH2, PRXQ (peroxiredoxin Q), TRX (thioredoxin), NTR (NADPH-dependent thioredoxin reductase), HSP90 (heat shock protein 90), CAT (catalase), SAL1 (inositol polyphosphate 1-phosphatase), midasin AAA ATPase 1 (MDN1), NIN/RPN12 binding protein (NOB1), nonsense-mediated decay 3 (NMD3), and eukaryotic translation initiation factor 2 (eIF2).

Under high light conditions exceeding photosynthetic capacity, production of harmful ROS is unavoidable associated with electron transport in the photosystems. Excess electron leakage to molecular oxygen and incomplete water oxidation produce singlet oxygen (^1^O_2_), superoxide (O_2_^⋅⁣–^), hydrogen peroxide (H_2_O_2_), and hydroxyl radical (HO^⋅^) (Pospíšil, 2016). This triggers a signaling cascade communicating the chloroplast state to the nucleus termed retrograde signaling that ultimately induces the expression of nuclear genes. The evolution of this system has played a central role in plant terrestralization (Zhao et al., 2019; Calderon and Strand, 2021). Retrograde signaling induced by ROS is dependent on executer (EX, *kfl00184_0040*), whose gene expression was not affected in our experiment, and on the FtsH2 protease (*kfl00201_0150*) strongly activated in our study (Dogra et al., 2017, 2019b; Kim, 2020). Indeed, response to oxidative stress was one of the most significant GO term in our functional enrichment analysis over the activated genes in a response to high light treatment in *K. nitens*. More than twofold activation was detected in genes encoding chloroplast targeted antioxidant enzymes such as catalase (CAT, *kfl01057_0030*) and peroxiredoxins Q (PRXQ, *kfl00014_0230* and *kfl00014_0250*) that, together with carotenoids such as zeaxanthin, contribute to ROS scavenging (Pinnola and Bassi, 2018). Under these conditions proteins suffer oxidative damage specifically but not limited to the active thiol groups of cysteine residues, which are oxidized to disulfide bonds (Cejudo et al., 2021). This produces major modifications in protein structure that can lead to misfolding and loss of function. The accumulation in the chloroplast of aberrant misfolded proteins also contributes to initiate retrograde signaling (Dogra et al., 2019a). In this respect, activation was identified for genes such as *kfl00120_0050* and *kfl00573_0030* encoding several thioredoxin-disulfide reductases (TRX) and *kfl00021_0420* corresponding to NADPH-dependent thioredoxin reductase (NTR). These enzymes constitute a system involved in oxidative damage avoidance by supplying reducing power to reductases repairing oxidized proteins (Vieira Dos Santos and Rey, 2006; Figure 4). Moreover, we found the activation of multiple chloroplast targeted chaperones, co-chaperones and chaperonins that would contribute to restore misfolded proteins, such as heat shock proteins 90 and 101 (HSP90, *kfl00002_0530* and HSP101, *kfl00387_0020*); chloroplast chaperonin 60 alpha and beta subunits (CPN60A, *kfl00113_0150* and CPN60B, *kfl00076_0150*), specifically involved in Rubisco correct folding (Zhao and Liu, 2018), and chloroplast GrpE involved in correct oligomerization of the photosynthesis-related light harvesting complex II (LHCII) in *Arabidopsis* (de Luna-Valdez et al., 2019). An example of a protein that suffers severe oxidative damage under high light stress is the D1 protein (PsbA) located at PSII reaction center. Specific tryptophan residues undergo oxidation in this protein triggering protein repair mechanisms (Dogra et al., 2019a). It has been shown that the aminoacids trypthophan and histidine easily suffer photooxidation (Huvaere and Skibsted, 2009). Recent studies have shown accumulation of tryptophan and phenylalanine under several environmental conditions as high light, drought, and temperature stress in Embryophyta (Galili et al., 2016). In this respect, our analysis shows that similar accumulation of the amino acids tryptophan, phenylalanine, and histidine takes place in a response to high light stress in Charophyta, like *K. nitens*, Supplementary Table 2.

Concomitant to the activation of protein repair mechanisms we found significant activation of ribosome biogenesis and cytoplasmic translation initiation (Figure 1). For example, genes encoding nuclear enzymes involved in 18S, 40S, and 60S rRNA biogenesis (UTP15 *kfl00593_0050*, NOB1 *kfl00104_0280*, and MDN1 *kfl00198_0260*) were strongly activated. Similarly, the genes CRM1 (*kfl00518_0070*) and NMD3 (*kfl00006_0030*), encoding nuclear export systems of these ribosomal components, were also detected as more than twofold activated. Additional activation was identified, for instance, in genes corresponding to eukaryotic translation initiation factor 2 (eIF2 *kfl00434_0020*) and 3 (eIF3 *kfl00078_0290*) (Figure 4). These strongly activated processes are required for *de novo* protein synthesis and, together with the previously described protein repair mechanisms, constitute part of the response to high light in *K. nitens*, contributing to maintain proteome homeostasis under this stress.

Besides, the retrograde signaling pathways induced by ROS and aberrant misfolded proteins discussed above, there exists another pathway regulated by the accumulation of 3′-phosphoadenosine-5′-phosphate (PAP). The inositol polyphosphate 1-phosphatase (SAL1) removes PAP preventing its accumulation. The gene encoding this enzyme *kfl00096_0240* was twofold repressed indicating a possible accumulation of PAP and an activation of the SAL1-PAP retrograde signaling pathway, as a response to high light intensity in *K. nitens*.

### Cyclic Electron Flow Is Strongly Induced as a Response to High Light

**FIGURE 5:**
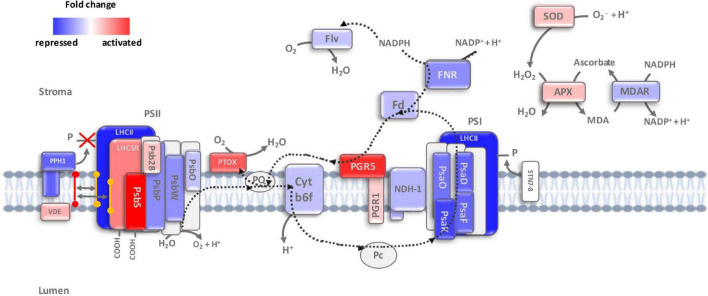
Electron flow through photosystems and non-photochemical quenching mechanisms dissipating excess light energy. Expression fold change under high light compared to low light is represented for genes corresponding to the different proteins involved in linear and cyclic electron flow, NPQ, state transitions and water-water cycle: violaxanthin de-epoxidase (VDE), light harvesting complex II (LHCII), LHC-like stress related (LHCSR) protein, photosystem II subunit S (PsbS), photosystem II subunit 28 (Psb28), photosystem II subunit P (PsbP), photosystem II subunit W (PsbW), photosystem II subunit O (PsbO), plastid terminal oxidase (PTOX), plastoquinone (PQ), cytochrome b6f (Cytb6f), proton gradient regulation 5 (PGR5), PGR-like 1 (PRGL1), NADH dehydrogenase-like complex (NDH), photosystem I subunit O (PsaO), photosystem I subunit K (PsaK), photosystem I subunit D (PsaD), photosystem I subunit F (PsaF), photosystem I subunit D (PsaD), photosystem I subunit F (PsaF), ferredoxin (Fd), ferredoxin-NADP-reductase (FNR), flavodiiron proteins (Flv), protein phosphatase 1 (PPH1), state transition 7/8 (STN7/8), superoxide dismutase (SOD), ascorbate peroxidase (APX) and monodehydroascorbate radical reductase (MDAR). Red represents strong activation whereas blue stands for strong repression under high light.

Photosynthetic electron flow operates in two modes, linear and cyclic (Suorsa, 2015). Our transcriptomic analysis unveiled an antagonist regulation of these two systems as a response to high light in *K. nitens*. The major route of electron transport in oxygenic photosynthesis is linear electron flow (LEF) initiated from water by harvesting sunlight and transferring excitation energy by LHCII to PSII and then through cytochrome b6f (Cytb6f) to photosystem I (PSI) and NADP+ toward the Calvin–Benson cycle. Proteins encoded by repressed genes during the response to high light in *K. nitens* were significantly associated with both photosystems resulting in a strong repression of photosynthesis and hexose biosynthesis (Figure 1). For example, we found more than twofold repression for genes corresponding to the LHCII protein LHCB1 (*kfl00098_0080*); to the PSII proteins PsbP (*kfl00239_0120*) and PsbW (*kfl00638_0030*); to the Cytb6f protein PetC (*kfl00433_0020*); and to the PSI proteins PsaD (*kfl00193_0150*) and PsaO (*kfl00283_0090*) (Figure 5). Funneling electrons from PSI to the Calvin–Benson cycle we found similarly repressed genes encoding ferredoxin-NADP-reductase (FNR, *kfl00169_0050*) and ferredoxin (Fd, *kfl00017_0060*). Correspondingly, gene repression was identified for all the enzymes involved in CO_2_ assimilation from the Calvin–Benson cycle. This indicates a strong repression of LEF in *K. nitens* under high light stress overexciting photosystems and producing electron excess that would damage them. Certainly, we observed lower values for PSII maximal efficiency (F_v_/F_m_) under high light 0.49, when compared to low light 0.66. As described in the previous section an excess of electrons at the PSI acceptor side results in reduction of molecular oxygen and generation of superoxide (O_2_^⋅⁣–^). The Mehler reaction or water–water cycle removes this harmful anion radical. In our transcriptomic analysis, we detected gene activation for enzymes in this cycle as superoxide dismutase (SOD, *kfl00631_0030*), converting O_2_^⋅⁣–^ to H_2_O_2_, and ascorbate peroxidase (APX, *kfl00460_0010*), which scavenges H_2_O_2_ with the aid of ascorbate to produce H_2_O and monodehydroascorbate radical (MDA). In turn, MDA is reduced by the MDA reductase (MDAR, *kfl00196_0050*) (Cardol et al., 2011). Nonetheless, the corresponding gene was found underexpressed in high light when compared to low light conditions. Flavodiiron proteins (Flv, *kfl00041_0060*) constitute another enzymatic system involved in the water–water cycle photoreducing O_2_ to H_2_O. Although Flv genes can be found in Charophyta genomes (Chaux et al., 2017) no activation was identified suggesting their expression is not related to the response to high light in *K. nitens* (Figure 5).

**FIGURE 6:**
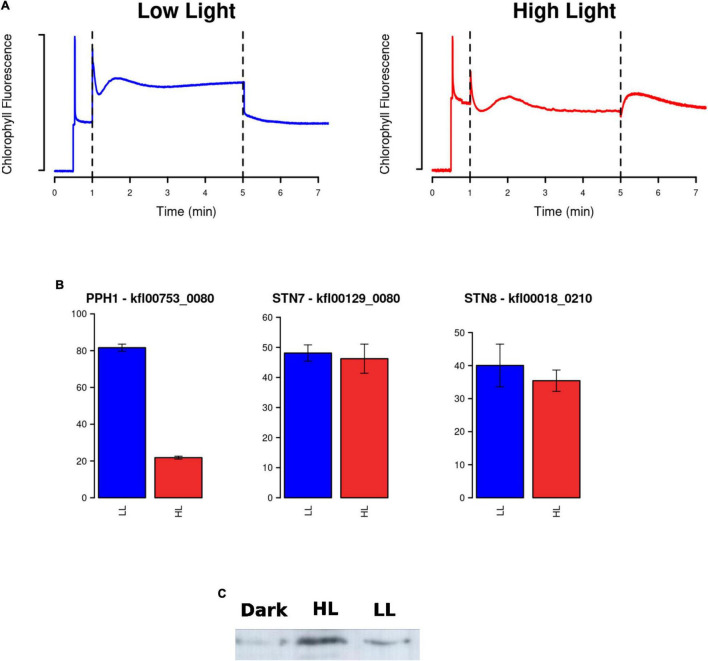
Measurement of cyclic electron flow (CEF), gene expression of enzymes involved in state transitions and identification of the response in PsbS protein abundance to light intensity. **(A)** Dark adapted *Klebsormidium nitens* cultures exposed to low light (blue) or high light (red) were subjected to actinic light after the first minute. CEF activity was detected after 5 min when actinic light was turn off and a clear transient increase in chlorophyll fluorescence was observed only for the high light cultures (red) using pulse-amplitude-modulation fluorometry. **(B)** Barplots representing gene expression measured in FPKM (Fragments per Kilobase of exon and million of Mapped reads) under high light in red (HL) and low light in blue (LL) for enzymes involved in state transitions: protein phosphatase 1 (PPH1) involved in dephosphorylation of light harvesting complex II (LHCII) associating it with photosystem II (PSII) and kinases State Transition 7/8 (STN7/8) involved in phosphorylation of LHCII associating it with photosystem I (PSI). **(C)** Identification of the PsbS protein abundance response using Western blotting with anti-PsbS antibody. Almost no PsbS protein was detected from cultures maintained under constant dark for 24 h (dark), whereas for cultures under high light (HL) greater PsbS protein abundance was observed when compared to low light (LL).

In contrast to LEF, our transcriptomic analysis unveiled a strong activation in CEF around PSI. In this route, electrons are recycled from Fd back to plastoquinone (PQ), creating a transthylakoid proton gradient, and leading to the production of only ATP (Figure 5). Indeed, we detected CEF activity as a clear transient increase in chlorophyll fluorescence after turning off actinic light by PAM in *K. nitens* cultures under high light that was not observed under low light (Figure 6). CEF has been identified as an essential regulatory process governing light acclimation and photosystems protection from ROS after high light exposure in Chlorophyta and Embryophyta (Iwai et al., 2010; Ma et al., 2021). Two different CEF pathways have been identified based on their different sensitivity to antimycin (Ravenel et al., 1994). The antimycin-insensitive CEF pathway is dependent on the NADH dehydrogenase-like complex (NDH). Even though this is the major pathway in cyanobacteria (Miller et al., 2021) and it plays crucial roles at low light intensity in Embryophyta such as rice (Yamori et al., 2015) and *Marchantia polymorpha* (Ueda et al., 2012), Ndh genes have disappeared from most Chlorophyta species including *Chlamydomonas*, *Chlorella*, *Scenedesmus*, and *Ostreococcus*. Nonetheless, Ndh genes are present in Charophytic genomes as *Chara* and *Mesostigma* (Peltier et al., 2010). Specifically, in *K. nitens*, several Ndh isoforms have been detected (Hori et al., 2014). However, all the Ndh subunits encoded by nuclear genes such as Ndh M/N/O (*kfl00053_0440*, *kfl00564_0070*, and *kfl00414_0120*) presented strong downregulation under high light (Figure 5). This suggests that the major CEF pathway in *K. nitens* under this stress is not dependent on the Ndh complex. In the antimycin-sensitive CEF pathway, the proteins proton gradient regulation 5 (PGR5, *kfl00020_0020*) and PGR-Like 1 (PRGL1, *kfl00342_0140*) are essential components. Genes corresponding to these proteins have been identified across Chlorophyta, Charophyta, and Embryophyta (Peltier et al., 2010; Hori et al., 2014; Yamamoto and Shikanai, 2019) which supports their relevance in the physiology of photosynthetic organisms. Although PGR5 plays a pivotal role in CEF, its function is not fully characterized. PGRL1 is a transmembrane protein with an iron cofactor, which is probably responsible for electron transfer from Fd to PQ (Ma et al., 2021). Both proteins PGR5 and PGRL1 need to operate jointly for efficient CEF to take place. Our analysis unveiled a strong activation of 5-fold in the gene encoding PGR5 and a mild activation of 1.24-fold in the gene for PGRL1 indicating that this is the major CEF pathway in response to high light in *K. nitens*.

The redistribution of excitation energy between PSII and PSI by reversible phosphorylation of LHCII, known as state transitions, plays an important role in light response in plants (Mekala et al., 2015). In state I, LHCII is phosphorylated by the kinases State Transition 7/8 (STN7/8, *kfl00129_0080* and *kfl00018_0210*) separated from PSII and adhered to PSI. In state II, LHCII is dephosphorylated by protein phosphatase 1 (PPH1, *kfl00753_0080*), and moved back to PSII. In our transcriptomic data, we found a strong repression greater than threefold in the gene encoding PPH1 and no significant changes in the genes for STN7/8 (Figure 6). This suggests that, in a response to high light, *K. nitens* locks photosystems in state I maintaining LHCII bound to PSI which would protect PSII from excess light and allocate light energy to PSI further enhancing CEF.

Another system contributing to adjust the redox poise of the photosynthetic electron transport chain tuning the ratios between LEF and CEF is constituted by the concerted operation between plastid terminal oxidase (PTOX, *kfl00009_0280*) and NADH dehydrogenase (Rumeau et al., 2007). Our transcriptomic analysis showed 3.5-fold upregulation of the corresponding gene for PTOX in high light cultures similar to a common adaptation strategy in marine phytoplankton to high light conditions (Cardol et al., 2008). This system has also been proposed to act as a safety valve during photosynthesis, preventing over reduction of the PQ pool during light stress (Niyogi, 2000).

### Photosystem II Subunit S and LHC-Like Stress Related Systems Are Simultaneously Activated Under High Light

Non-photochemical quenching plays an essential role in photoprotection dissipating excessive absorbed light energy as heat. Two different proteins for NPQ activation are known in Chlorophyta and Embryophyta, namely, the LHC-like stress related (LHCSR) protein and the PsbS. These two systems induce NPQ through different molecular mechanisms. LHCSRs are grouped under the so-called stress-induced chlorophyll-binding proteins (Dittami et al., 2010). The abundance of LHCSR increases under high light just as NPQ is induced (Peers et al., 2009). LHCSR homologs have been shown to be involved in NPQ in diatoms (Bailleul et al., 2010; Zhu et al., 2010). LHCSR binds pigments and is capable of efficiently dissipate excitation energy as heat. PsbS induces a reorganization of the photosynthetic apparatus by interfering with the formation of aggregates of thylakoid membrane proteins, thus allowing easy exchange and incorporation of xanthophyll cycle pigments into such structures. The structures formed in the presence of violaxanthin are characterized by minimized dissipation of excitation energy, whereas the structures formed in the presence of zeaxanthin show enhanced excitation quenching (Welc et al., 2021). It has been reported that NPQ relies mainly on PsbS in Embryophyta (Li et al., 2000) whereas in Chlorophyta the major role is played by LHCSR (Peers et al., 2009). Our study aims at contributing to the elucidation of these two systems in Charophyta as *K. nitens*.

Our transcriptomic analysis identified a strong overexpression of both systems under high light when compared to low light in *K. nitens*. Specifically, we detected a 3-fold activation of the gene *kfl00478_0030* corresponding to LHCSR and a massive upregulation of 114-fold in the gene encoding PsbS, *kfl00093_0070* (Figure 5). Although the PsbS transcript has been detected previously in *K. nitens*, the identification of the corresponding protein remained elusive (Hori et al., 2014). Accumulation of the PsbS protein has only been detected in Charophyta, such as *Zygnema* and *Mesotaenium* (Gerotto and Morosinotto, 2013) and so, it was discussed that the PsbS system is only operational in late and not in early Charophyta as *K. nitens*. In contrast to these previous negative results, we were able to detect the protein PsbS in *K. nitens* protein extracts using Western blotting (Figure 6). We observed almost no detectable PsbS protein from cultures maintained under constant dark for 24 h whereas for cultures under low light a band corresponding to PsbS was detected whose level was increased in cultures under high light supporting a response of this system to increasing levels of light intensity (Figure 6).

Our results support the fact that, as a response to high light, both systems based on LHCSR and PsbS are strongly activated for efficient NPQ in early Charophyta as *K. nitens*. A similar response has been described for the Bryophyta *Physcomitrium patens* (Gerotto et al., 2012), which diverged from vascular plants early after land colonization. In this specie, both systems are also active, contributing to efficient NPQ. This suggests the co-existence of these two NPQ mechanisms from early Charophyta to Bryophyta during plant evolution, before the emergence of vascular land plants that eventually lost LHCSR while retaining PsbS instead (Pinnola, 2019).

### Genes Present Only in Streptophyta Are Induced as a Response to High Light Intensity

**FIGURE 7:**
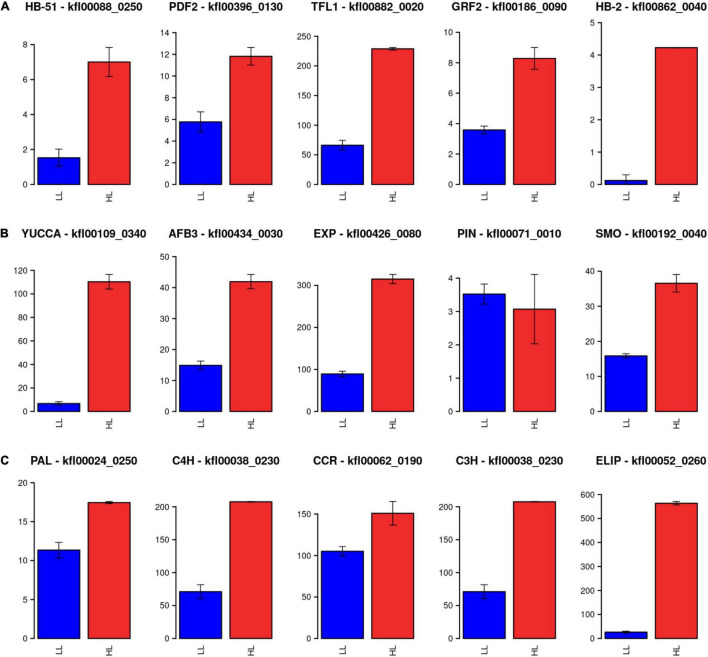
Expression level for Streptophyta exclusive genes. **(A)** Barplots representing gene expression measured in FPKM (Fragments per Kilobase of exon and million of Mapped reads) under high light in red (HL) and low light in blue (LL) for potential homolog genes of transcription factors regulating floral transition in *Arabidopsis*: Homeobox 51 (HB-51), Protodermal Factor 2 (PDF2), and Terminal Flower 1 (TFL1). **(B)** Barplots representing gene expression measured in FPKM (Fragments per Kilobase of exon and million of Mapped reads) under high light in red (HL) and low light in blue (LL) for potential homolog genes involved in auxin biosynthesis, sensing, and response in *Arabidopsis*: YUCCA, auxin signaling F-box 3 (AFB3), expansin (EXP), PIN, and sterol 4-alpha-methyl-oxidase (SMO). **(C)** Barplots representing gene expression measured in FPKM (Fragments per Kilobase of exon and million of Mapped reads) under high light in red (HL) and low light in blue (LL) for potential homolog genes involved in the phenylpropanoid pathway: phenylalanine ammonia-lyase (PAL), cinnamate 4-hydroxylase (C4H), cinnamoyl-CoA reductase (CCR), and 4-coumarate 3-hydroxylase (C3H). The high activation of early light induced protein (ELIP) is also shown.

**FIGURE 8:**
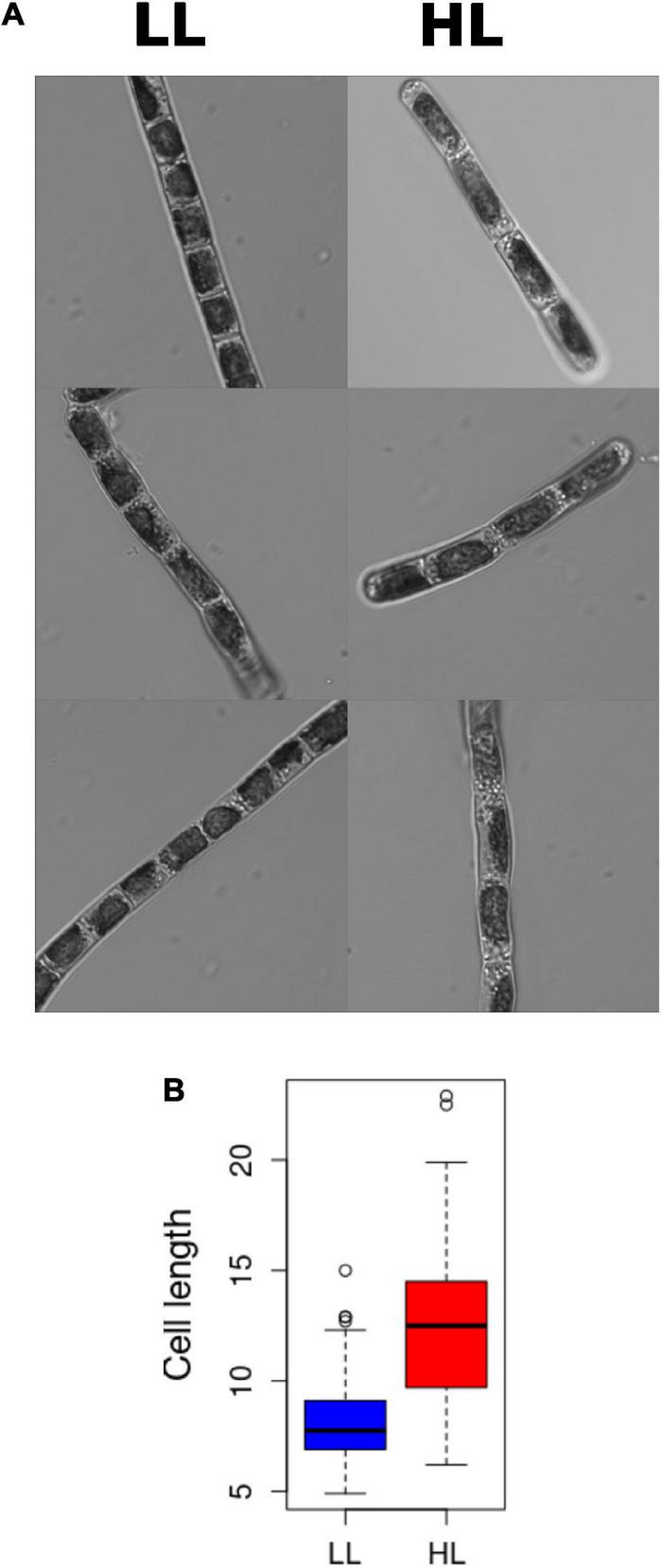
*Klebsormidium nitens* cell elongation under high light. **(A)** Confocal images of *Klebsormidium nitens* cells under low light (LL) on the left and high light (HL) on the right. **(B)** Boxplot representing the significative increase in cell elongation under high light (HL) in red when compared to low light (LL) in blue.

Based on sequence similarity, specific genes only found in Streptophyta were identified in *K. nitens*, as those without a significant homolog in Chlorophyta genomes (Hori et al., 2014). These genes could have played important roles in the adaptation of Charophyta to terrestrial environments, promoting land colonization by Streptophyta. According to this classification, we found 70 specific Streptophyta activated genes corresponding to 10% of the whole activated transcriptome in *K. nitens*. Several genes with significant sequence homology to transcription factors involved in development in Embryophyta were identified. For example, potential homolog genes of transcription factors regulating floral transition in *Arabidopsis* were found activated as *kfl00088_0250* (Homeobox 51), *kfl00396_0130* (Protodermal Factor 2), and *kfl00882_0020* (Terminal Flower 1). Similarly, we identified, as significantly activated, the genes *kfl00186_0090* and *kfl00862_0040* potential homologs of the transcription factors growth regulating factor 2 (grf2) and homeobox 2 (HB-2), respectively (Figure 7). These are regulators of cell growth, expansion and proliferation as a response to phytohormones in *Arabidopsis* (He et al., 2020). Genomic studies have unveiled certain types of primitive land plant signaling pathways for phytohormone response in *K. nitens* (Holzinger and Becker, 2015; Holzinger and Pichrtová, 2016). These primitive phytohormone systems may be involved in various responses to harsh environmental stresses on land in this early Charophyta (Hori et al., 2014). Our metabolomic analysis identified the presence of the phytohormones salicylic acid (SA), gamma-aminobutyric acid (GABA), ABA, indole-3-carboxylic acid (ICA), and IAA in *K. nitens* cultures, Supplementary Table 2. None of these phytohormones changed significantly except IAA with a threefold increase under high light (Figure 2). Auxins are synthesized from tryptophan through a pathway where the protein YUCCA plays a central role. As described previously, our metabolomic analysis found a significant threefold increase in tryptophan abundance under high light that could be related to the increase in IAA content. Moreover, the gene *kfl00109_0340* encoding YUCCA exhibited a large 16-fold increase in expression in a response to high light in *K. nitens*. Other Streptophyta specific genes involved in auxin sensing and response such as the receptor *kfl00434_0030* (auxin signaling F-box 3, AFB3) and *kfl00426_0080* (expansin, EXP) were also significantly activated under high light (Figure 7). Nevertheless, no significant change was observed in the gene *kfl00071_0010* encoding the single PIN auxin transporter protein identified in *K. nitens*. However, the expression of the related gene *kfl00192_0040*, encoding a sterol 4-alpha-methyl-oxidase (SMO), was also increased under high light. It has been suggested that SMO enzymes affect the polar localization of PIN altering developmental processes in Embryophyta (Zhang and Li, 2016). Auxins has been described to induce cell elongation in plants (Ma and Li, 2019). Although no phenotypic change was apparent after 3 h of high light treatment, we could observe significant cell elongations (*p*-vale 1.3 × 10^–11^) in *K. nitens* filaments after 72 h (Figure 8). Nonetheless, the observed cell elongation could be produced by the arrest in cell cycle identified in our functional enrichment analysis (Figure 1) rather than on the activation of IAA responsive genes such as EXP (Ohtaka et al., 2017).

The phenylpropanoid pathway was considered to be an important Embryophyta specific source for metabolites relevant to environmental stresses. Nonetheless, candidate homologous genes codifying for enzymes acting at different steps in this pathway have recently been identified in *K. nitens* (de Vries et al., 2021). Concomitant to an increase in the aromatic amino acid phenylalanine, input to this pathway (Figure 2), some of these genes were found activated under high light stress in our study (Figure 7). Specifically, one of the candidate genes encoding phenylalanine ammonia-lyase (PAL) *kfl00024_0250* was 1.53-fold activated. Although no clear cinnamate 4-hydroxylase (C4H) homolog has been determined in *K. nitens* one of the candidates *kfl00038_0230* was 3-fold activated. Similarly, candidates for 4-coumarate 3-hydroxylase (C3H), *kfl00038_0230*, and cinnamoyl-CoA reductase (CCR), *kfl00062_0190*, were 2.92- and 1.43-fold activated, respectively.

Early light induced proteins (ELIPs) were thought to be Embryophyta specific stress responsive mechanisms (Adamska et al., 1992). Nonetheless, ELIPs have been found activated in Charophyta under abiotic stresses such as heat and desiccation (Holzinger et al., 2014; De Vries et al., 2018; Rippin et al., 2019b; de Vries et al., 2020). In our study, genes codifying for ELIPs were among the most activated ones under high light stress, for example, *kfl00052_0260* was more than 21-fold activated (Figure 7).

## Conclusion

*Klebsormidium nitens*, as a representative of early Charophyta, exhibit the co-existence of high light response mechanisms that are also detected in Chlorophyta and Embryophyta. Assuming that these systems have not independently arisen in *K. nitens*, our results support a model by which the streptophyte ancestor of land plants conserved features of its chlorophyte relatives and also developed new signaling responses to high light that promoted the transition to land and the emergence of Embryophyta. *K. nitens* possesses a tight and fast chloroplast retrograde signaling, possibly mediated by ROS and the SAL1-PAP pathways, as suggested by the downregulation of the *SAL1* gene. This system, after only 3 h of high light, induces gene expression and accumulation of specific metabolites to overcome this stress, especially relevant in terrestrial environments. Precisely, mechanisms common to Chlorophyta and Embryophyta are induced such as the xanthophyll cycle, with VDE activation and ZEP repression, leading to the accumulation of zeaxanthin; and protein repair mechanisms based on the NTR-TRX-PRX system. Further photoprotective mechanisms were identified such as the downregulation of LEF and the upregulation of CEF, with the specific activation of PGR5 and repression of Ndh components, and PPH1 locking LHCII in state I associated with PSI. More interestingly, the simultaneous strong activation of NPQ mechanisms specific to Chlorophyta, as LHCSR, and to Embryophyta, as PsbS, were detected. Finally, specific Embryophyta systems for auxin synthesis, sensing, and response were activated leading to an increase in auxin content with the concomitant accumulation of amino acids such as tryptophan, histidine, and phenylalanine. Moreover, specific genes in the phenylpropanoid pathway and ELIPs were also activated in our study. All these systems could have been major facilitators for plants conquest of terrestrial environments since they would have enabled an adaptation of land plants algal ancestors to high light, one of the major stressors for Charophyta in terrestrial habitats.

## Data Availability Statement

The raw and processed datasets generated and analyzed in this study can be found in the Gene Expression Omnibus data base identified with the accession number GSE198330 (https://www.ncbi.nlm.nih.gov/geo/). Raw sequencing data can be accessed from the Sequence Read Archive (https://www.ncbi.nlm.nih.gov/sra) identified with the accession number PRJNA814727. Raw UPLC/MS metabolomic data can be accessed from the database MetaboLights (https://www.ebi.ac.uk/metabolights/) using the identifier MTBLS4467.

## Author Contributions

MM-P and MEG-G performed the experiment, collected the samples, processed them for metabolomic analysis, and generated and analyzed the confocal microscopy images. ES-P and AR-L extracted and purified the RNA for sequencing. ES-P, AR-L, and FR-C performed the transcriptomic and metabolomic data analysis, visualization of the results, and carried out PAM measurements and analysis. IC, ES-P, and AR-L performed the protein extraction, SDS-PAGE, and Western blotting analysis. FR-C and MG-G designed the experiments, supervised the research, interpreted the results, and wrote the manuscript. All authors read and approved the manuscript.

## Conflict of Interest

The authors declare that the research was conducted in the absence of any commercial or financial relationships that could be construed as a potential conflict of interest.

## Publisher’s Note

All claims expressed in this article are solely those of the authors and do not necessarily represent those of their affiliated organizations, or those of the publisher, the editors and the reviewers. Any product that may be evaluated in this article, or claim that may be made by its manufacturer, is not guaranteed or endorsed by the publisher.

## Footnotes

1
https://greennetwork.us.es/AlgaeFUN/
