# Supplementary figures and images for: Transcriptomic and Metabolomic Response to High Light in the Charophyte Alga Klebsormidium nitens
Source: Front Plant Sci. 2022 May 6;13:855243. doi: 10.3389/fpls.2022.855243 (PMC9121098; doi:10.3389/fpls.2022.855243)

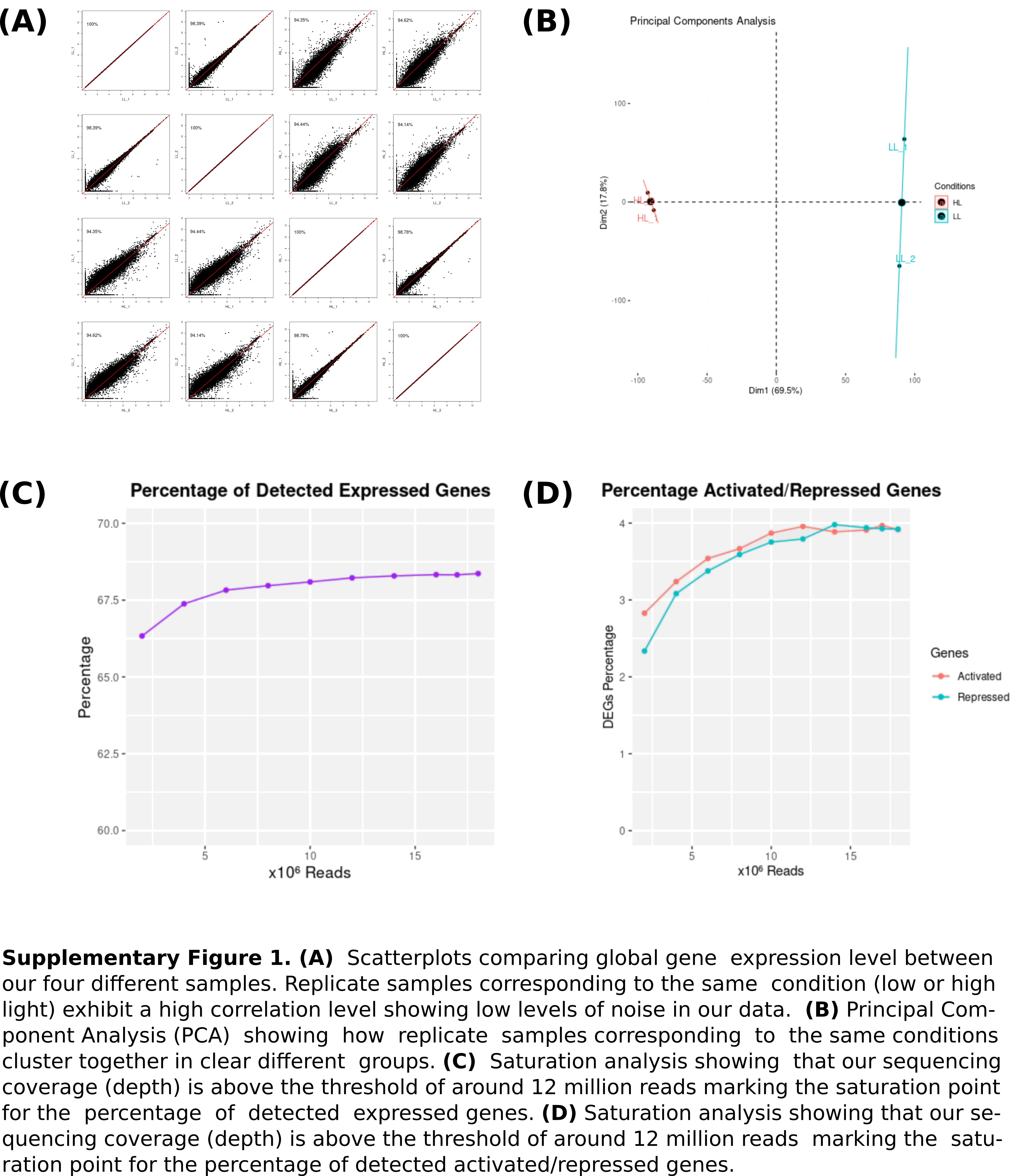

Supplement: Supplementary file 3 [file Image_1.PNG]
